# Whole-Genome Sequencing of Multidrug-Resistant Escherichia coli Strains Harboring the *mcr-1* Gene, Isolated from Seawater of the Algiers Coast in Algeria

**DOI:** 10.1128/MRA.00638-19

**Published:** 2019-08-22

**Authors:** M. Berrazeg, A. Deriet, S. C. J. De Keersmaecker, B. Verhaegen, K. Vanneste, N. Botteldoorn, N. H. C. Roosens, F. Mouffok, R. Drali

**Affiliations:** a Laboratoire de Microbiologie, Institut Pasteur d'Algérie, Antenne d'Oran, Oran, Algeria; b Département de Biologie, Faculté des Sciences de la Nature et de la Vie, Université d’Oran 1, Oran, Algeria; c Unité environnement, Laboratoire de Bactériologie des Aliments, Eaux et Environnement, Institut Pasteur d'Algérie, Alger, Algeria; d Sciensano, Transversal Activities in Applied Genomics, Brussels, Belgium; e Sciensano, Service Food Borne Pathogens, Brussels, Belgium; Georgia Institute of Technology

## Abstract

Colistin resistance has emerged worldwide and is threatening the treatment efficacy of multiresistant Escherichia coli strains in humans and animals. Here, we communicate the whole-genome sequencing (WGS) of two colistin-resistant E. coli strains, M49 and M78, with genomes sizes of 4,947,168 and 5,178,716 bp, respectively, isolated from seawaters of the Algiers coast.

## ANNOUNCEMENT

Colistin is a last-resort drug for the treatment of infections caused by multidrug-resistant Escherichia coli. In 2015, Liu et al. (1) described for the first time a colistin resistance mechanism mediated by the *mcr-1* gene situated on a transferable plasmid. Since then, several reports have indicated that *mcr-1* has silently spread worldwide since 1980 (2). During the summer of 2016, environmental sampling at 62 different beaches on the Algiers coast was carried out. An amount of 500 μl of each sample was incubated on 5 ml of brain heart infusion broth (BHIB) for 24 h at 37°C, and then positive cultures (turbidity higher than 0.5 McFarland standard) were inoculated on Hecktoen agar supplemented with 2 μg/ml colistin. Two E. coli strains (M49 from West Algiers and M78 from East Algiers) were confirmed by PCR and Sanger sequencing to contain the *mcr-1* gene (3), for which we present the genome sequencing here. Genomic DNA was extracted using a Genomic-tip 20/G (Qiagen), following the manufacturer’s instructions. A paired-end 2 × 250-bp sequencing run was performed using an Illumina MiSeq system. The Nextera XT DNA library preparation kit was used to construct libraries from the extracted DNA. Raw sequence reads were trimmed using Trimmomatic v0.36.4 with the following options: trailing, 10; leading, 10; slidingwindow, 4:20; and minlen, 40 (4). Assembly was carried out using SPAdes v1.3.1, with default settings (5). Annotation of the assembly was done using Prokka rapid prokaryotic genome annotation v1.11 with default settings (6). Antimicrobial resistance (AMR) gene occurrence was investigated with ResFinder v3.1, with default settings (https://cge.cbs.dtu.dk/services/ResFinder/). The presence of plasmid replicons was explored using PlasmidFinder v2.0, with default settings (https://cge.cbs.dtu.dk/services/PlasmidFinder/), and the serotype was determined using SerotypeFinder v2.0, with default settings (https://cge.cbs.dtu.dk/services/SerotypeFinder/).

A total of 762,976 and 1,057,562 reads of 250 bp were obtained for strains M49 and M78, respectively. Strain M49, with a total genome size of 4,947,168 bp, harbored 15 resistance genes belonging to eight different families of antibiotics, aminoglycosides [*aac(*3*)-IId*, *aadA1*, *aadA2*, *aph(3′)-Ia*, *aph(3ʺ)-Ib*, and *aph(*6*)-Id*], beta-lactams (*bla*_TEM-1B_), colistin (*mcr-1.5*), macrolides [*mph*(A)], phenicols (*cml*), sulfonamide (*sul1* and *sul3*), tetracycline [*tet*(A)], and trimethoprim (*dfrA1* and *dfrA14*). The genes *aadA1*, *aadA2*, *sul1*, and *tet*(A) were carried by an IncFII-type plasmid that exhibits 80% identity with the K-12 plasmid pDM0133 (GenBank accession number KJ170699), whereas the *mcr-1.5* gene was localized on an Incl2-type plasmid (GenBank accession number MG825369) with 100% identity. Two other plasmids, namely, IncFIB (GenBank accession number KJ484628; 75.35% identity) and Col156 (GenBank accession number AP017615; 54.16% identity) were detected in this strain. The strain M78, with a total genome size of 5,178,716 bp, harbored 10 resistance genes belonging to eight different families of antibiotics, namely, aminoglycoside [*aac(3)-IIa* and *aph(3′)-Ia*], beta-lactam (*bla*_TEM-1A_), colistin (*mcr-1.1*), fluoroquinolone (*qnrS1*), macrolide [*mdf*(A) and *mph*(A)], sulphonamide (*sul3*), tetracycline [*tet*(A)], and trimethoprim (*dfrA14*). The genes *mcr-1.1* and *sul1* were localized on the IncHI2A-type plasmid (GenBank accession number MH208235; 95.97% identity), whereas *bla*_TEM-1A_ and *aph(3′)-Ia* were contained on the IncX1-type plasmid (GenBank accession number KU254580; 95.12% identity). The plasmids IncI1 (GenBank accession number CP024152; 92.02% identity) and IncI2 (GenBank accession number CP024148; 96.77% identity) were detected in M78 as well.

Our results demonstrate that seawater is a potential reservoir of colistin-resistant E. coli, which possibly also carries other resistance genes. Seawater might be spreading a cocktail of multiple resistances, posing a worrisome threat to public health.

### Data availability.

The genomic sequences are deposited in the NCBI Sequence Read Archive and NCBI GenBank (BioProject number PRJNA540702) and are listed in Table 1.

**TABLE 1:** Metadata of the two Escherichia coli strains, M49 and M78

Strain	Subtype	Serotype	No. of plasmids	Genome size (bp)	No. of CDS^*a*^	No. of contigs	No. of contigs >1,000 bp	GC content (%)	*N*_50_ (bp)	No. of tRNAs	Genome coverage (×)	GenBank accession no.	SRA accession no.
M49	708	O:78 H:9	4	4,947,168	4,666	247	118	50.55	119,930	63	26	GCA_006348985	SRR9099604
M78	21	O:174 H:32	5	5,178,716	4,899	288	123	50.29	108,253	65	20	GCA_006348965	SRR9099605

a CDS, coding DNA sequences.
